# Organic solution advanced spray-dried microparticulate dry powder of doxycycline hyclate for lung delivery

**DOI:** 10.1038/s41598-026-39198-9

**Published:** 2026-03-09

**Authors:** Hanan Alameddin, Wafaa Alabsi, Basanth Babu Eedara, Neftali Ortega Alarcon, Richard L De Armond, Saif Mashaqi, Sairam Parthasarathy, Roberto Guzman, Heidi M. Mansour

**Affiliations:** 1 The University of ArizonaR. Ken Coit College of PharmacySkaggs Pharmaceutical Sciences Center, Tucson, AZ USA; 2 Center for Translational Science, Florida International University, Port St. Lucie, FL USA; 3 Robert Stempel College of Public Health and Social Work, Departmentof EnvironmentalHealthSciences, Florida International University, Weston, FL USA; 4 Present Address: Transpire Bio Inc, 2945 W Corporate Lakes Blvd Suite A, Miami, FL 33331 USA; 5 The University of Arizona Health Sciences Center for Sleep Circadian and Neurosciences Research, Tucson, AZ USA; 6 College of Engineering, DepartmentsofChemicalandEnvironmentald-Engineering, The University of Arizona, Tucson, AZ USA; 7 College of Engineering, DepartmentsofBiomedicalEngineering, The University of Arizona, Tucson, AZ USA; 8 Department of Medicine, DivisionofTranslationalandRegenerative Medicine, The University of Arizona College of Medicine, Tucson, AZ USA; 9 Department of Cell and Molecular Medicine, Florida International University, HerbertWertheimCollegeofMedicine, Miami, FL USA

**Keywords:** Obstructive Sleep Apnea (OSA), Doxycycline, Spray drying, Dry Powder Inhaler (DPI), Lung inflammation, Aerosolization, Biotechnology, Drug discovery, Materials science, Nanoscience and technology

## Abstract

Obstructive sleep apnea (OSA) is a common sleep disorder characterized by the upper airway collapse, leading to interrupted breathing and reduced oxygen levels during sleep. This condition often results in chronic inflammation and is associated with various long-term health problems. To address OSA-related inflammation, an FDA-approved drug, Doxycycline, was formulated in this study as a dry powder inhaler due to its favorable anti-inflammatory properties that was tested in subsequent cellular and animal studies of OSA. This comprehensive and systematic study aimed to develop inhalable excipient-free spray-dried (SD) one-component drug powders of Doxycycline**.** Advanced organic solution spray-drying in closed mode, with three different feed pump rates (10%, 50%, and 100%), was used to design and produce Doxycycline microparticles in the solid state successfully. The SD Doxycycline formulations comprised hollow, spherical, dimpled particles (<2 μm). The solid-state characterization of SD formulations confirmed the amorphous nature of Doxycycline after spray drying with a glass transition temperature between 82.73 °C and 89.29 °C. However, compared to the raw drug, residual water content was higher in the SD formulation, ranging from 6.4 ± 0.02% w/w in SD Doxycycline at the low feed pump rate to 6.79 ± 0.06% w/w in SD Doxycycline at the high feed pump rate. SD formulations showed good aerosolization behavior with a Respirable Fraction (RF) of >60% with NeoHaler inhaler device (63.19 ± 7.59 to 68.53 ± 3.73%) while HandiHaler showed lower RF (39.61± 0.57 to 53.90 ± 8.03%). The fine particle fraction (FPF) was higher in the NeoHaler group (24.91 ± 1.17 to 36.72 ± 2.01%) compared to HandiHaler (19.61 ± 5.41 to 33.76 ± 5.21%). This could be explained by the difference in median aerodynamic diameter (8.21 ± 2.68 to 4.69± 1.27 μm) in the NeoHaler group compared to (3.07 ± 0.36 to 3.67 ± 0.59 μm) HandiHaler group. Furthermore, *in vitro* cell viability as a function of lung cell type and drug dose showed 10 µM of Doxycycline as a safe concentration for the A549, H358, H441, and Calu-3 epithelial-like immortal human cell lines. Inhalable dry powder formulation of Doxycycline could offer new treatment options for patients suffering from sleep apnea.

## Introduction

Obstructive sleep apnea (OSA) is the most common sleep-related breathing disorder, characterized by obstructive apneas, hypopneas, or other respiratory effort-related arousals caused by repetitive upper airway collapse during sleep^1^. OSA affects 9% to 38% of the general adult population^2^. Several studies have found a relationship between OSA and systemic inflammation^3^. Furthermore, inflammatory biomarkers such as C- reactive protein (CRP), interleukin-6 (IL-6), tumor necrosis-alpha (TNF-α), and matrix metalloproteinases have been seen in the pathogenesis of intermittent hypoxia that is caused by OSA^4,5^^.^ There is currently no medication that is specifically approved by the United States-Food and Drug Administration (US-FDA) for the treatment of OSA. Doxycycline is a semisynthetic bacteriostatic broad-spectrum antibiotic in the therapeutic class of tetracycline antibiotics. It was first developed for clinical use in early 1960 and was granted US-FDA approval in 1967^6,7^. In addition to its bacteriostatic properties, Doxycycline possesses anti-inflammatory properties and has been used as an anti-inflammatory agent in different medical fields, such as dermatology and dentistry^8^. When used at lower dosage than required for bacteriostatic effect, Doxycycline interferes with the synthesis of inflammatory mediators such as phospholipid A2^9–11^. Several studies have shown that a subantimicrobial dose (40 mg/day) of Doxycycline is safe and effective for treating mild to severe papulopustular rosacea and chronic periodontitis^12^. Studies have also shown Doxycycline’s ability to inhibit additional inflammatory proteins, such as matrix metalloproteinases (MMPs)^13^. Furthermore, by targeting neutrophils and macrophages, Doxycycline also suppresses the release of inflammatory cytokines such as TNF-α, IL-1β, and IL-6 and plays a role in regulating the immune system^11,14^. For these reasons Doxycycline an excellent candidate to consider in treating conditions in which inflammation is involved in the pathophysiology, such as OSA-induced hypoxia^15^. Delivering potential drugs for the treatment of OSA, such as Doxycycline, through the pulmonary route offers several advantages. The lungs’ high surface area and blood supply offer several advantages for local and systemic drug delivery while avoiding the hepatic first-pass effect which affects drugs administered by the oral route of administration^16,17^.For instance, in 2008, Gueders et al. successfully demonstrated that Doxycycline delivered as aerosol decreased airway inflammation caused by allergens through modulating cytokine production and MMP activities in mice^18^. In this study, inhalable excipient-free drug powders composed of Doxycycline nanoparticles/microparticles for targeted pulmonary drug delivery as DPIs were designed and produced using advanced organic solution spray drying. Comprehensive physicochemical properties of the raw drug and spray-dried (SD) powders were carried out. To the authors’ knowledge, this is the first study to report these findings.

## Materials and methods

### Materials

Doxycycline Hyclate [C_22_H_24_N_2_O_8_ · HCl · 0.5H_2_O · 0.5CH_6_O, molecular weight (MW) 512.94 g/mole, purity ≥ 93.5%, (ChemDraw^®^ Professional version 16.0., CambridgeSoft, Cambridge, MA, USA)) was purchased from Sigma-Aldrich (Milwaukee, WI, USA). Ethanol (HPLC-grade, 99.9%) was purchased from Sigma-Aldrich (Milwaukee, WI, USA). AquaStar anhydrous methanol was obtained from EMD Millipore Corporation, an Affiliate of Merck (Darmstadt, Germany). Hydranal^®^ Coulomat AD was obtained from Honeywell Fluka (Muskegon, MI). Four epithelial-like immortal cell lines, A549, H358, H441 and Calu-3, representing different regions of the lung: A549 (lung adenocarcinoma alveolar Type I-derived epithelial cells, ATCC^®^ CCL-185TM), NCI- H358 (bronchioalveolar carcinoma cells, ATCC^®^ CRL-5807TM), H441 (lung adenocarcinoma alveolar Type II-derived epithelial cells ATCC® HTB174TM) and Calu-3 (human lung adenocarcinoma bronchial cells ATCC^®^ HTB-55TM) were purchased from the American Type Culture Collection ATCC^®^ (Manassas, VA, USA). The Eagle’s minimum essential medium (EMEM) was also purchased from ATCC^®^ (Manassas, VA, USA). RPMI 1640 medium was purchased from Thermo Fisher Scientific (Waltham, MA, USA) *,* Advanced 1×, fetal bovine serum (FBS), Pen-Strep, and Fungizone^®^, were obtained from Gibco^®^ by Life Technologies (Thermo Fisher Scientific Inc., Waltham, MA, USA).

### Methods

#### Powder production

Using a Büchi advanced mini spray dryer B-290 coupled with a Büchi inert loop B-295 and a high- performance cyclone (Büchi Labortechnik AG, Switzerland), dry powder particles of Doxycycline were produced under similar conditions to those used in previous study^19^. Nitrogen gas was used as the atomizing gas in a closed-mode spray drying process. A stainless-steel nozzle with a diameter of 0.7 mm was employed for atomization, and a 0.1% w/v feed solution of Doxycycline Hyclate dissolved in methanol was used. The spray drying process was carried out at a flow rate of 600 L/h (50 mm Hg) for the atomization gas and an aspirator rate of 35 m^3^/h. Different spray drying feed pump rates corresponding to low, medium, and high feed pump rates of 3 mL/min (10%), 15 mL/min (50%), and 30 mL/min (100%), respectively, were used (Table 1). An inlet temperature (T_inlet_) of 150 °C was used for all feed pump rates. The resulting SD powders were collected in glass vials, sealed with parafilm, and stored in sealed glass desiccators over Indicating Drierite/Drierite™ desiccant at 2-8 °C under ambient humidity conditions.

**Table 1:** Spray drying conditions for 0.1% w/v Doxycycline Hyclate dissolved in methanol using organic solution advanced spray drying method in closed mode.

**Spray drying feed pump rate (%)**	**T**_inlet_ (°C)	**T**_outlet_ (°C)
10	150	80
50	150	71
100	150	62

#### Scanning electron microscopy

(SEM) Using similar conditions reported previously^20^; microscopic images were captured using an FEI Inspect S^®^ microscope (FEI, Brno, Czech Republic). Powder samples were adhered to aluminum stubs using double- sided adhesive carbon tabs (TedPella, Inc. Redding, CA, USA) and coated with a thin film of gold using the Hummer 6.2 sputtering system from Anatech (Union City, CA, USA) operated at 15 AC milliamperes with around 7 kV of voltage for 90 s. The electron beam with an accelerating voltage of 30 kV was used at a working distance between 12.4-15.4 mm.

#### Particle size distribution

As previously reported representative SEM micrographs^21,22^of each powder sample at 8,000× magnification were analyzed with ≥100 particles per powder sample having their diameter measured geometrically and the particle size distribution calculated statistically by SigmaScan Pro5.0.0 (Systat, San Jose, CA, USA)

#### X-ray Powder Diffraction (XRPD)

As previously reported^19^, crystallinity of the raw drug and SD formulations was assessed using a PANalytical X’pert^®^ diffractometer (PANalytical Inc., Westborough, MA, USA) equippedwith a programmable incident beam slit and an X’celerator detector at room temperature conditions. Powder samples were loaded on a zero-background silicon sample holder and then subjected to X-ray radiation using a Ni-filtered Cu Kα (45 kV, 40 Ma, and λ = 1.5444 Å) over an angular range of 5.0° to50.0° (2θ) with a scan rate of 2.00 theta-degrees/min.

#### Differential Scanning Calorimetry (DSC)

As previously reported^23^, thermotropic phase transition measurements (n = 3) were carried out using the TA Discovery DSC250^®^ (TA Instruments, New Castle, DE) equipped with T-Zero^®^ technology and an automated computer-controlled RSC-90 cooling system. Approximately 2-5 mg of sample was placed in hermetic anodized aluminum T-Zero^®^ DSC pans, which were hermetically sealed with theT-Zero^®^ hermetic sealer (TA Instruments). An empty hermetically sealed aluminum pan was used as the reference pan. UHP nitrogen was used as a purging gas at a rate of 50 mL/min. The sample was heated from 5 °C to 250 °C at a scanning rate of 5.00 °C/min and 20.00 °C/min. 

#### Hot-Stage Microscopy (HSM)

As previously reported^23^, powder samples were fixed on a glass slide and heated from 25.0 °C to 250.0 °C at a heating rate of 5.00 °C/min. Samples were visualized under cross-polarized light microscopy as a function of increasing temperature for the presence or absence of birefringence. The images were digitally captured using a Nikon Digital Sight 1000^®^ camera (Nikon, Tokyo, Japan) under 10× optical objective and 10× digital zoom.

#### Attenuated Total Reflectance-Fourier-Transform Infrared (ATR-FTIR) spectroscopy

Using similar conditions reported previously^24^ Nicolet IS50R FT-IR^®^ spectrometer (Waltham, MA, USA) equipped with an DTGS detector and a Thunderdome attenuated total reflectance (ATR) (Spectra-Tech, Oak Ridge, TN, USA) accessory with a germanium window was used for molecular fingerprinting by ATR-FTIR^25,26^.

Each spectrum was collected for 32 scans at a spectral resolution of 8 cm^−1^ over the wavenumber range of 4000–400 cm^−1^. Spectral data were processed using OMNIC Spectra Software (Thermo Scientific™,Waltham, Massachusetts).

#### Raman spectroscopy molecular fingerprinting and confocal Raman microscopy chemical imaging mapping

Using conditions previously reported^27^, Raman spectroscopy was used for molecular fingerprinting and Confocal Raman microscopy was used for chemical image mapping using a 785 nm excitation wavelength at 50× magnification objective on a Leica DM2700 optical microscope (Wetzlar, Germany). Raman spectral analysis was performed on raw drug and SD powders at three different powder spots. Three accumulations were performed for each spot using 10 s of detector exposure time per accumulation with spectral scanning from 50–4000 cm^−1^ (data shown only up to 2000 cm^−1^). Spectra were subjected to baseline correction before further analysis using Renishaw WiRE version 3.4 software. (Renishaw, Gloucestershire, United Kingdom).

#### Karl Fischer Titration (KFT)

The residual water content of powders was chemically quantified by coulometric Karl Fischer titration (KFT) using a TitroLine 7500 trace titrator (SI Analytics, Weilheim, Germany). Similar to conditions previously reported^21^, approximately 1–5 mg of powder was directly added to the titration cell containing Hydranal^®^ Coulomat AD reagent to measure water content in raw and SD powders.

#### In vitro aerosol dispersion performance

The Next Generation Impactor^TM^ (NGI^TM^) (NGI Model 170, MSP Corporation, Shoreview, MN, USA) was used to assess powder aerosolization (n=3), following USP Chapter <601> specifications on aerosol^28^. Two FDA-approved human DPI devices, HandiHaler^®^ (Boehringer Ingelheim, Ingelheim, Germany) and NeoHaler^®^ (Novartis AG, Stein, Switzerland) were utilized in this study. HandiHaler^®^ requires stronger inhalation to generate shear while NeoHaler^®^ (Novartis AG, Stein, Switzerland) utilizes medium shear stress. The NGI was connected to the Copley HCP5 high-capacity vacuum pump with a Copley TPK 2000 critical flow controller (Copley Scientific, Nottingham, UK) and attached to a stainless-steel induction port (i.e. USP throat) equipped with specialized stainless steel NGI gravimetric insert cups (MSP Corporation, Shoreview, MN, USA) at an airflow rate of 60 L/min. Three Quali-V clear HPMC size 3 inhalation grade capsules (Qualicaps, NC, USA) were individually weighed and each were filled with ~10 mg of powder. Each capsule was inserted into the aerosol device, and the device was tightly fitted to a mouthpiece attached to the induction port. The powder mass on each stage was determined gravimetrically using A/ E glass filter paper with diameters of 55 mm (PALL Corporation, Port Washington, New York, USA) and 75 mm (Advantec^®^, Japan). The *in vitro* aerodynamic parameters, emitted dose (defined as the amount of drug that is emitted from the inhaler and can be inhaled (ED)), fine particle fraction which is percentage of the fine particles emitted from the inhaler, (%FPF), mass median aerodynamic diameter (MMAD), geometric standard deviation (GSD), respirable fraction defined as the fraction of the powder particles that reach the site of action (%RF)^29^ were all calculated as follows

1 $$\text{Emitted Dose fraction }\left(\mathrm{ED\%}\right)= \frac{\mathrm{ED}}{\mathrm{TD}}\text{X }100\mathrm{\%}$$

2 $$\text{Fine Particle Fraction }\left(\mathrm{FPF\%}\right)=\frac{\mathrm{FPD}}{\mathrm{ED}}\text{X }100\mathrm{\%}$$

3 $$\text{Respirable Fraction }\left(\mathrm{RF\%}\right)=\frac{\mathrm{FPD}}{\mathrm{DD}}\text{ X }100\text{ \%}$$

#### In vitro human lung cell viability

The effects of Doxycycline Hyclate on cell viability as a function of drug dose and cell type was examined^22,23^. Four epithelial-like immortal cell lines, A549 (lung adenocarcinoma alveolar Type I-derived epithelial cells, ATCC^®^ CCL-185TM), NCI-H358 (bronchioalveolar carcinoma cells, ATCC^®^ CRL-5807TM), H441 (lung adenocarcinoma alveolar Type II- derived epithelial cells ATCC® HTB174TM) and Calu-3 (human lung adenocarcinoma bronchial cells ATCC^®^ HTB-55TM) were used. Upon reaching confluence, cells were seeded in 96- black well plates at a concentration of 5,000 cells/well and 100 μL/well. To ensure confluency, cells were incubated for 48 h to allow attachment to the surface of the plates and the subsequent formation of a monolayer. Raw powder of Doxycycline Hyclate was then dissolved in RPMI and EMEM media. Different drug concentrations of 0.1 -1000 μM were prepared by serial dilution. A volume of 100 μL of each drug solution at a given concentration was added to each well (n=6). Cells were incubated at 37 °C and 5% CO_2_ for 72 h. A Resazurin assay was performed by adding 20 μL of 20 μM resazurin sodium to each well followed by 4 h of incubation at 37 ^◦^C and 5% CO_2_. The fluorescence intensity of the metabolite (i.e. resorufin) produced by viable cells was measured at 544 nm excitation wavelength and 590 nm emission wavelength using Molecular Devices^®^ SpectraMax^®^ M3 Multi-Mode Microplate Reader (Sunnyvale, CA, USA)

4 $$\text{Relative Viability }\left(\mathrm{\%}\right)= \frac{\text{Sample Fluorescence Intensity}}{\text{Control Fluorescence Intensity}}\text{X }100\mathrm{\%}$$

#### Statistical analysis

All experiments were performed in triplicates (n = 3). The results were analyzed statistically using SigmaPlot^®^ 13 (SYSTAT Software, Inc., San Jose, CA) scientific software and expressed as mean ± standard deviation. As previously reported^30^ one-way analysis of variance (ANOVA) statistical method was used to compare the relative viability between cells exposed to the drug and control cells. A value of p < 0.05 was considered significant.

## Results

### Scanning Electron Microscopy (SEM) analysis: Morphological and Particle Size Characterization

**Fig. 1:**
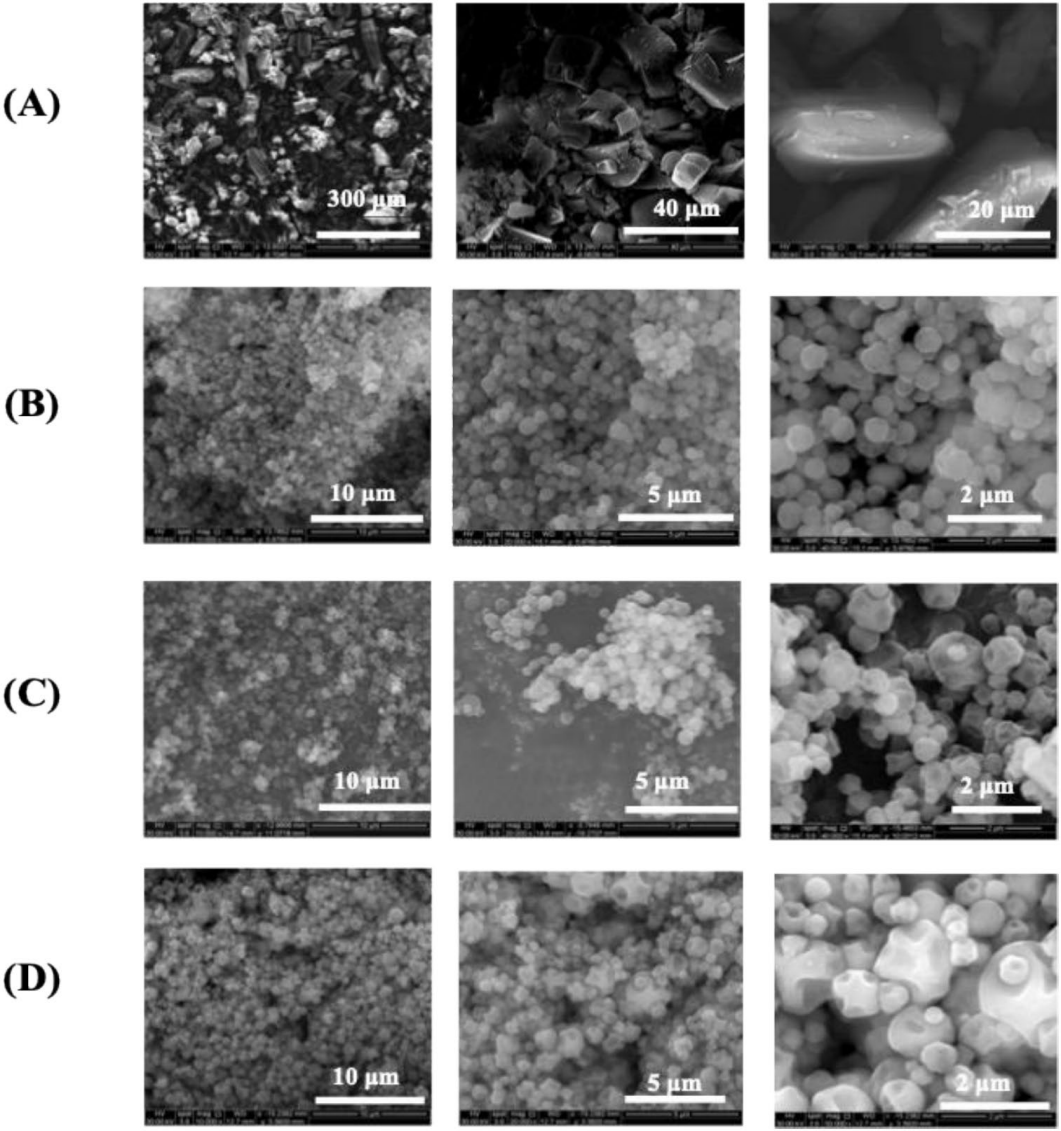
SEM micrographs of (**A**) Raw Doxycycline Hyclate (**B**) SD Doxycycline at 10% PR, (**C**) SD Doxycycline at 50%, (**D**) SD Doxycycline at 100% PR.

 SEM images showed a difference in particle shape, particle size, and surface morphology between raw drug and SD powders Figure 1. As seen in Figure 1A, raw drug particles appeared larger in size and irregular in shape with a rough surface morphology. In contrast, SD particles were discreet, small, spherical, smooth, and dimpled, as shown in Figure 1B-1D

### Particle size distribution: medium pump rate yields smallest particles

It is important to note that although lower feed pump rates generally produce smaller particles, this is not always the case. Several factors, including liquid viscosity, feed material characteristics, drying conditions, and collection methods can influence the final particle size and size distribution. In our study, particles produced at the medium feed pump rate yielded the smallest particle size, Table 2.

**Table 2:** Particle Sizing (n ≥ 100 particles).

**System composition**	**Mean size (µm) ± SD**	**Particle size range (µm)**
SD Doxycycline (10% PR)	0.633 ± 0.115	0.376-0.957
SD Doxycycline (50% PR)	0.514 ± 0.119	0.248-0.837
SD Doxycycline (100% PR)	1.028 ± 0.338	0.429-2.028

### XRPD patterns: crystallinity of raw drug vs. amorphus SD powder

**Fig. 2:**
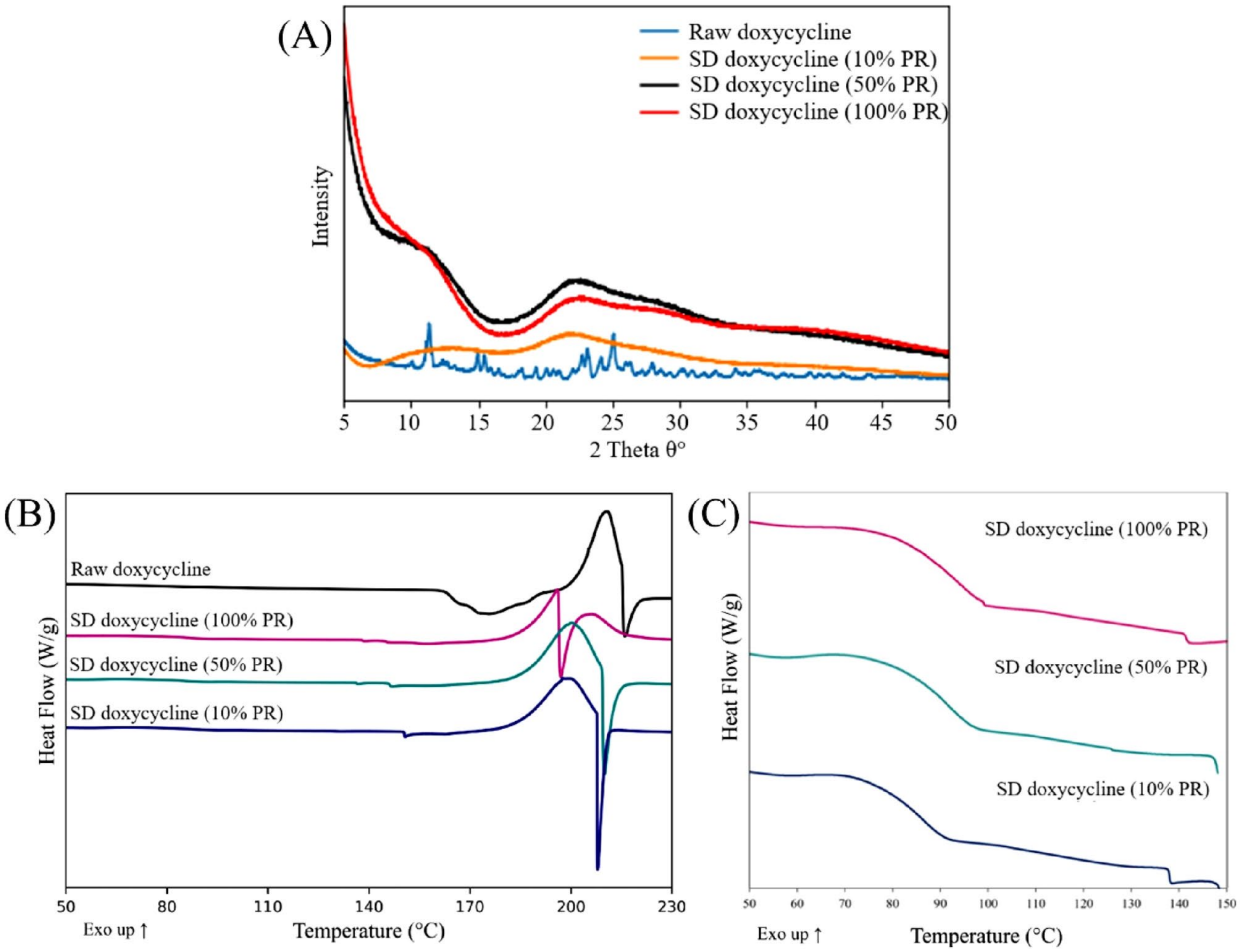
(**A**) XRPD diffractograms for raw and SD Doxycycline powders, (**B**) DSC thermograms of raw and SD Doxycycline powders when heated at 5.00°C/minute (slow heating scan rate), (**C**) DSC thermograms of raw and SD Doxycycline powders when heated at 20.00°C/minute (fast heating scan rate).

The XRPD diffraction pattern of raw Doxycycline Hyclate (Figure 2A) showed multiple sharp and intense peaks throughout, particularly at 12° (2θ) and 23-25° (2θ) representing the presence of long-range molecular order confirming the crystalline nature of the raw drug. In contrast, the diffraction patterns of the SD powders (Figure 2A) showed the absence of sharp peaks, indicating the loss of long-range molecular order and the amorphous nature of the SD powders.

### Differential Scanning Calorimetry (DSC): glass transition in SD powder

As shown in Figures 2B, 2C and Table 3, an endothermic molecular order-to-disorder thermodynamic transition peak was seen at ~175 °C for the raw drug heated at 5.00 °C/min and 20.00 °C/min (to detect first-order thermodynamic phase transitions) possibly due to the removal of the bound water molecule from the Hyclate salt. This was followed by an exothermic molecular disorder-to-order transition peak at ~ 210 °C followed by an endothermic molecular-order-to-disorder peak at ~216 °C.

**Table 3:** Thermal analysis of raw and SD Doxycycline powders heated at 5.00 °C/min heating scan rate (mean ± standard deviation, n = 3).

**Formulation**	**T**_**peak**_** (°C)**	**Enthalpy (J/g)**	**T**_**g**_** (°C)**	**Delta C**_**p**_** (J/g)**	**Texotherm (°C)**	**Tendotherm (°C)**
Raw Doxycycline	175.32 ± 1.78	51.81 ± 16.93	-	-	211.12 ± 1.24	216.36 ± 3.96
SD Doxycycline (10% PR)	-	-	87.50 ± 1.41	0.52 ± 0.2	198.86 ± 1.71	208.15 ± 0.33
SD Doxycycline (50% PR)	-	-	82.96 ± 0.23	0.48 ± 0.11	199.44 ± 0.76	209.25 ± 2.85
SD Doxycycline (100% PR)	-	-	87.02 ± 2.27	0.61 ± 0.04	199.96 ± 3.66	207.46 ± 5.68

As seen in Figure 2C** and **Table 4, all SD Doxycycline powder samples when heated at a fast-heating scan rate of 20.00 °C/min displayed a distinct baseline shift that is characteristic of T_g_. This occurred in the temperature range of 82 °C-90 °C, indicating a second-order kinetic phase transition. The T_g_ values of all SD powders were above a biological temperature of 37 °C. The melting points of all SD powders were similar

**Table 4:** Thermal analysis of SD Doxycycline Hyclate powders heated at 20.00 °C/minute heating scan rate (mean ± standard deviation, n = 3).

**System composition**	**T**_**peak**_** (°C)**	**Enthalpy (J/g)**	**T**_**g**_** (°C)**	**Delta C**_**p**_** (J/g)**	**Texotherm (°C)**	**Tendotherm (°C)**
SD Doxycycline (10% PR)	-	-	88.79 ± 0.39	0.61 ± 0.07	223.88 ± 1.88	173.33 ±1.41
SD Doxycycline (50% PR)	-	-	87.50 ± 1.41	0.52± 0.20	198.86 ± 1.71	208.15 ± 0.33
SD Doxycycline (100% PR)	-	-	84.50 ± 0.98	0.69 ± 0.07	227.96 ± 1.72	166.21±3.34

### Hot-Stage Microscopy (HSM): lack of birefringence in SD powder

**Fig. 3:**
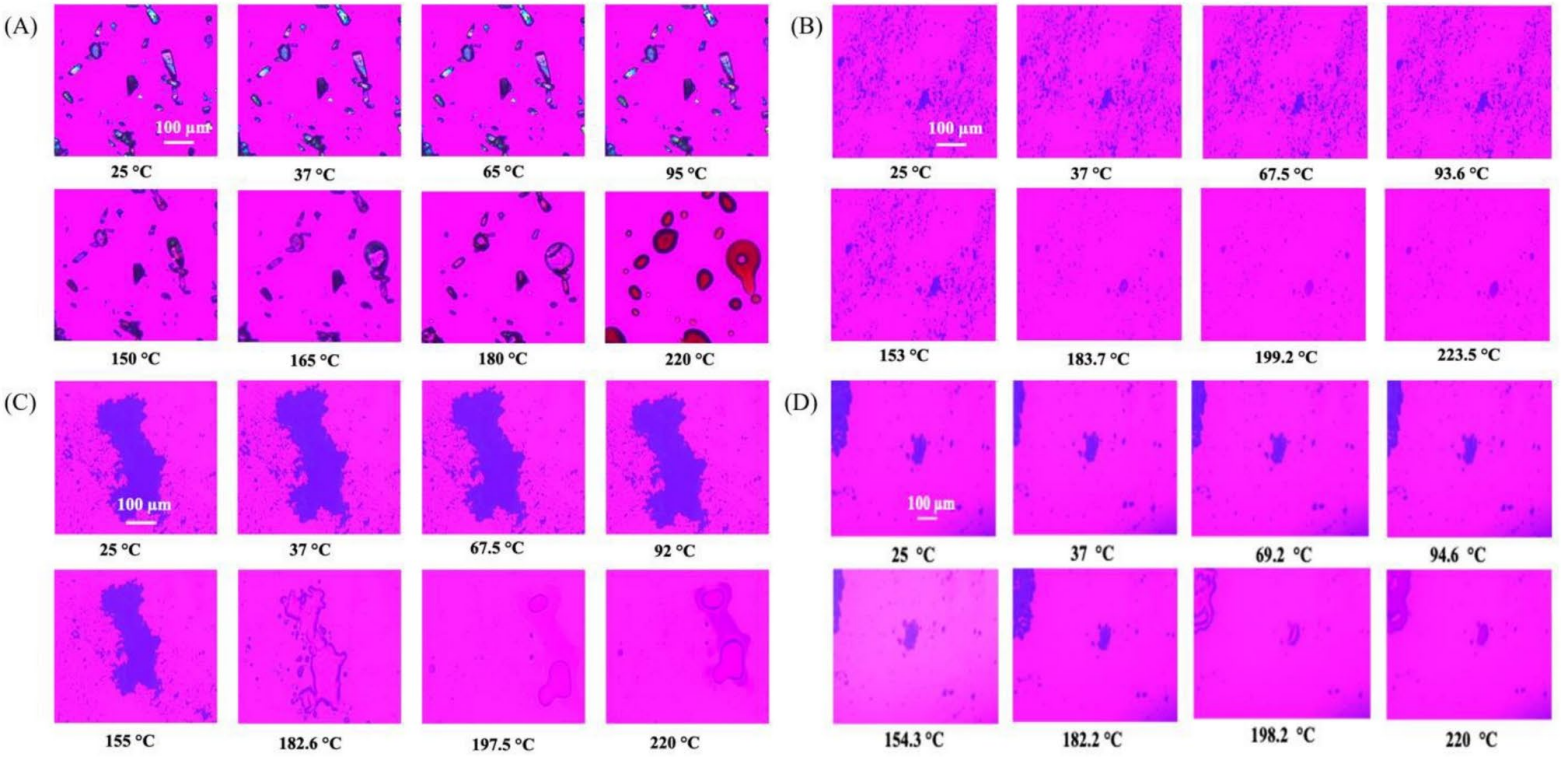
HSM micrographs of (**A**) raw Doxycycline Hyclate (**B**) SD Doxycycline at 10% PR, (**C**) SD Doxycycline at 50%, (**D**) SD Doxycycline at 100% PR.

As shown in Figure 3A**,** raw drug powder exhibited birefringence. SD powders (10%, 50% and 100% respectively) showed a lack of birefringence at all temperatures (Figure 3B - 3D). Furthermore, there was no observable difference in birefringence observed at room temperature (25 °C) and at a biological (37 °C).

### Effect of spray drying and feed pump rate on ATR-FTIR spectroscopy

**Fig. 4:**
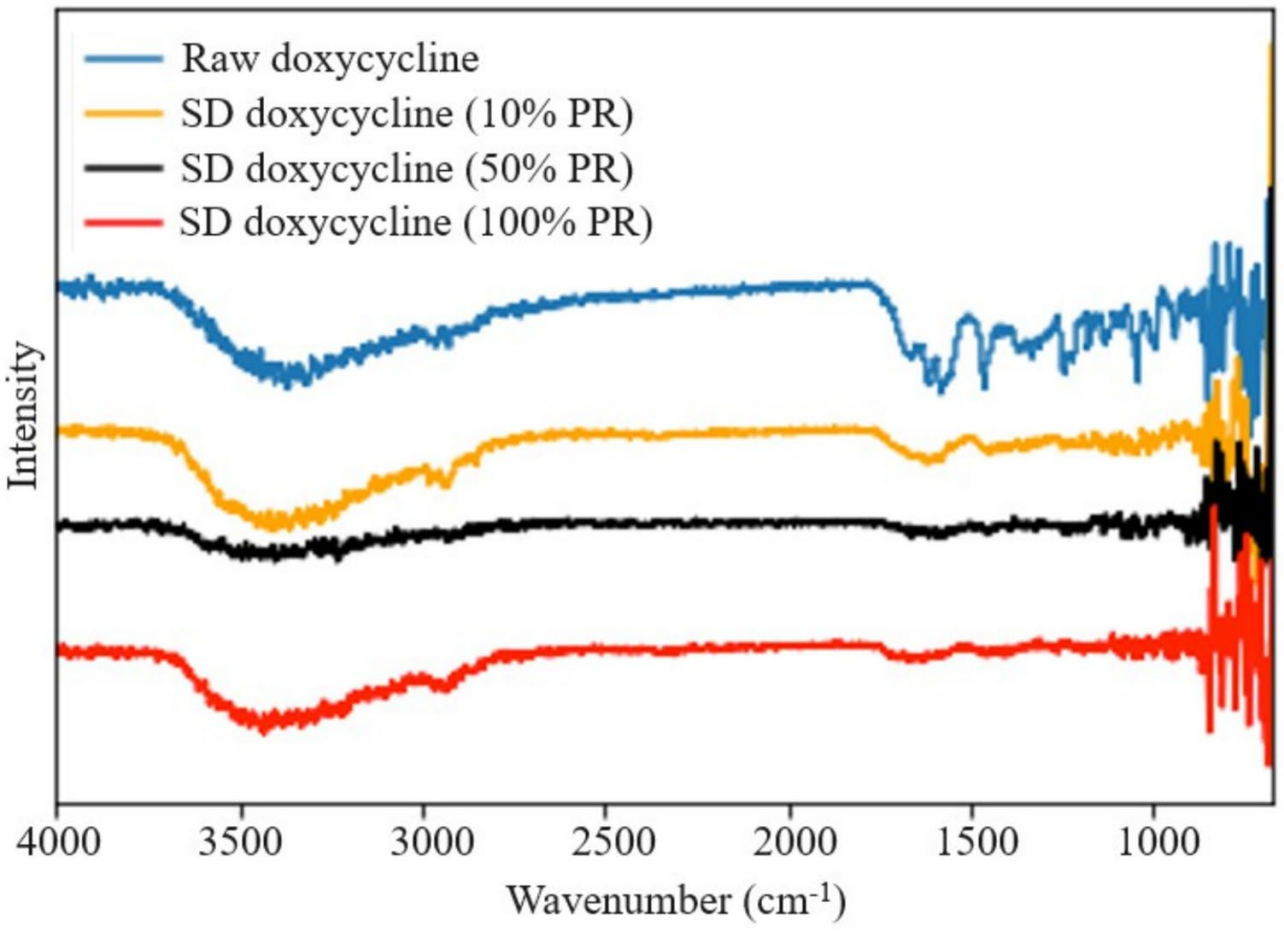
ATR-FTIR spectroscopy of raw and SD Doxycycline powders.

The ATR-FTIR spectra are shown in Figure 4 The ATR-FTIR spectrum for raw Doxycycline Hyclate powder was in agreement with previously published literature and showed a broad peak at 3500 cm^−1^ corresponding to NH and OH. Multiple peaks between 1600-1700 cm^−1^ correspond to C=O and C=C stretches for aromatic rings, amide band, and carboxylic acid band respectively

As seen in Figure 4, the ATR-FTIR spectra displayed a broad peak at 3500 cm_−1_ for the 100% and 10% SD powders, while other peaks were absent except for a peak of 1600 cm^-1^_−1_at 100% feed pump rate.

### Assessment of sample homogeneity using raman spectroscopy and confocal raman microscopy

**Fig. 5:**
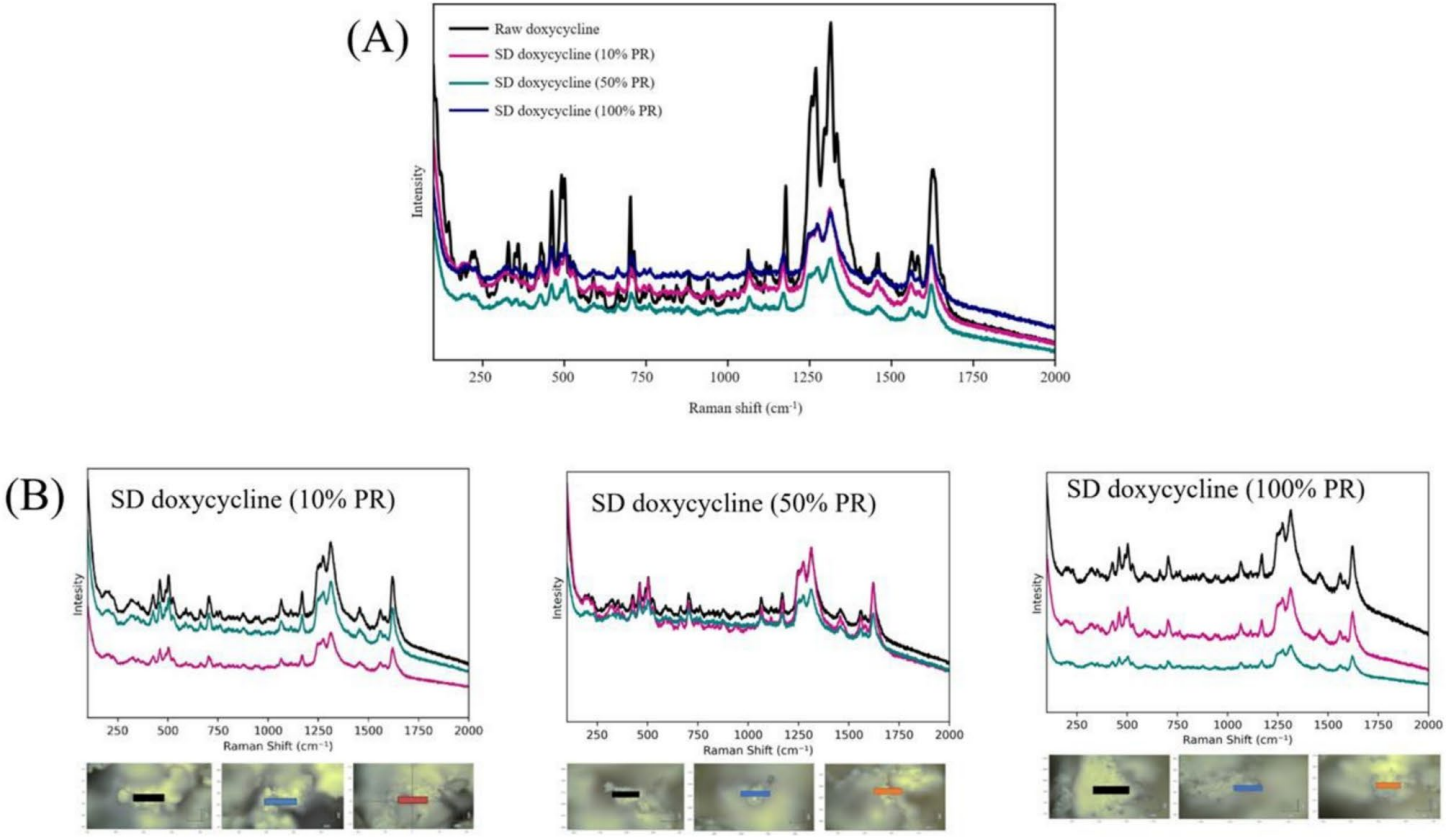
(**A**); Raman spectroscopy molecular fingerprinting of raw and SD Doxycycline powders (**B**; Confocal Raman microscopy chemical imaging of SD Doxycycline 10%, 50% and 100% PR.

All the samples exhibited a lack of crystallinity after spray drying compared to the raw drug which showed high crystallinity before spray drying. Figure 5A shows the molecular fingerprinting of raw Doxycycline and SD powders using Raman spectroscopy. Several Raman shifts were observed; the first one was in the range of 400-500 cm^-1^_−1_, followed by shift in a range of 600-700 cm^-1^_−1_. Additional Raman shifts were observed at 1200-1300 cm−1 and 1600-1700 cm^-1^_−1_. Using confocal Raman microscopy for chemical imagemapping as shown in Figure 5B. Three different spots were viewed for Raman spectra for each SD Doxycycline powder. Raman spectra were identical across these spots for the same sample, confirming homogeneity of the SD powders.

### Karl Fischer titration: water content comparison between SD powder and raw doxycycline hyclate

The residual water content was relatively low for all systems, as listed in Table 5. The SD powders had higher residual water content than raw drug powder. In addition, the spray drying feed pump rate affected the residual water content: the high spray drying feed pump rate resulted in the highest residual water content. In contrast, the lowest spray drying feed pump rate resulted in the lowest residual water content for all the SD powders.

**Table 5:** Residual water content measured by Karl Fischer coulometric titration for raw and SD Doxycycline powders (mean ± standard deviation, n = 3).

**Powder composition**	**Residual water content (% w/w)**
Raw Doxycycline	2.83 ± 0.19
SD Doxycycline (10% PR)	6.4 ± 0.02
SD Doxycycline (50% PR)	6.63 ± 0.14
SD Doxycycline (100% PR)	6.79 ± 0.06

### In vitro aerosol dispersion performance in Neohaler vs Handihaler^®^

**Fig. 6:**
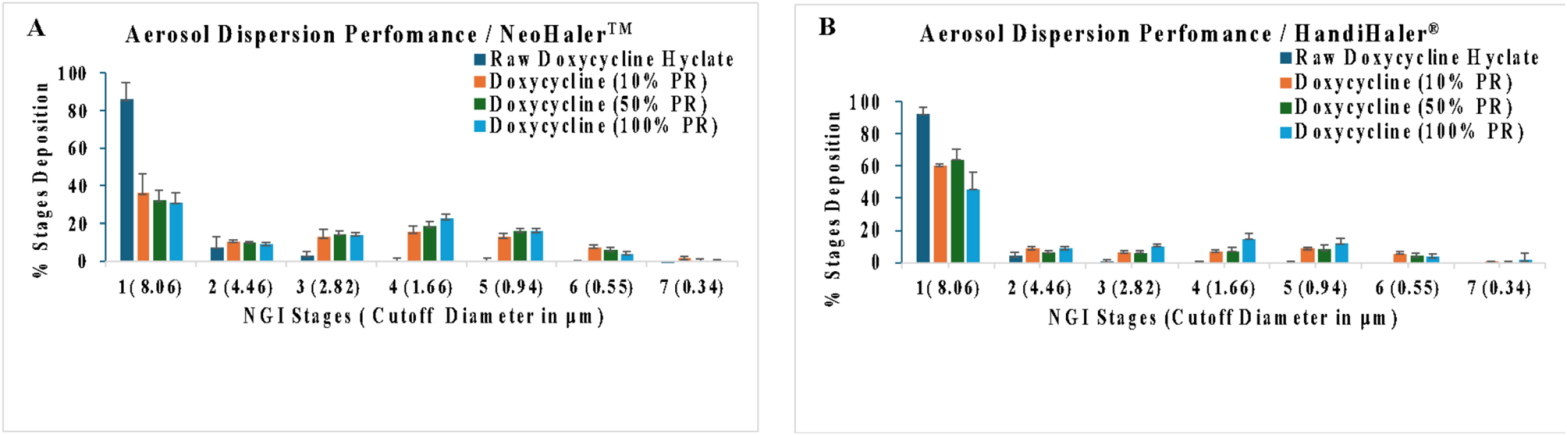
In vitro aerosol dispersion performance of raw and spray dried (SD) Doxycycline powders using the Next Generation Impactor™ (NGI™) at a flow rate of 60 L/min with the human DPI devices, (**A**) NeoHaler™, and (**B**) HandiHaler^®^.

For the raw drug, most of the powder was deposited on NGI stage 1. The SD powders exhibited measurable deposition on all NGI stages, as shown in Figure 6 and Table 6. Overall, raw Doxycycline powder showed inferior aerosol dispersion properties (FPF%, RF%, and MMAD) which is not suitable for pulmonary inhalation aerosol delivery. However, ED values were above 88% for all systems, including the raw drug. For SD formulations, the FPF values ranged between ~14 and 39% using both devices. There was variations in RF values between both DPI devices. Specifically, the RF values were lower with the HandiHaler, compared to the NeoHaler, which ranged between 30–62% and 56–72%, respectively. The MMAD was ~3 -11 μm with the HandiHaler and ~3- 4 μm with the NeoHaler.

**Table 6:** *In vitro* aerosol dispersion performance as DPIs (n = 3, mean ± standard deviation). PR- feed pump rate; ED- emitted dose; FPF- fine particle fraction; RF- respirable fraction; MMAD-mass median aerodynamic diameter; GSD- geometric standard deviation

**Formulation**	**Inhaler device**	**ED (%)**	**FPF (%)**	**RF (%)**	**MMAD (μm)**	**GSD**
Raw Doxycycline		101.88 ± 3.69	3.638 ± 1.32	7.077 ± 2.86	81.11 ± 40.46	4.96 ± 2.55
SD Doxycycline (10% PR)	HandiHaler	98.67 ± 4.16	23.59 ± 0.29	39.61± 0.57	6.71 ± 0.96	3.97 ± 0.17
SD Doxycycline (50% PR)		93.32 ± 5.95	19.61 ± 5.41	35.51 ± 5.20	8.21 ± 2.68	3.98 ± 0.83
SD Doxycycline (100% PR)		97.69 ± 2.12	33.76 ± 5.21	53.90 ± 8.03	4.69± 1.27	3.37 ± 0.78
Raw Doxycycline		101.26 ± 1.22	4.41 ± 1.17	13.34 ± 6.77	31.18±9.05	3.06 ± 0.16
SD Doxycycline (10% PR)	NeoHaler	97.28 ± 0.13	24.91 ± 1.17	63.19 ± 7.59	3.67 ± 0.59	2.90 ± 0.21
SD Doxycycline (50% PR)		100.52 ± 4.28	28.04 ± 1.22	67.33 ± 4.08	3.22 ± 0.29	2.40 ± 0.08
SD Doxycycline (100% PR)		99.23 ± 2.21	36.72 ± 2.01	68.53 ± 3.73	3.07 ± 0.36	2.13 ± 0.14

### Assessment of biocompatibility and in vitro human lung cell viability: evaluating safetyrelated to drug concentration 

**Fig. 7:**
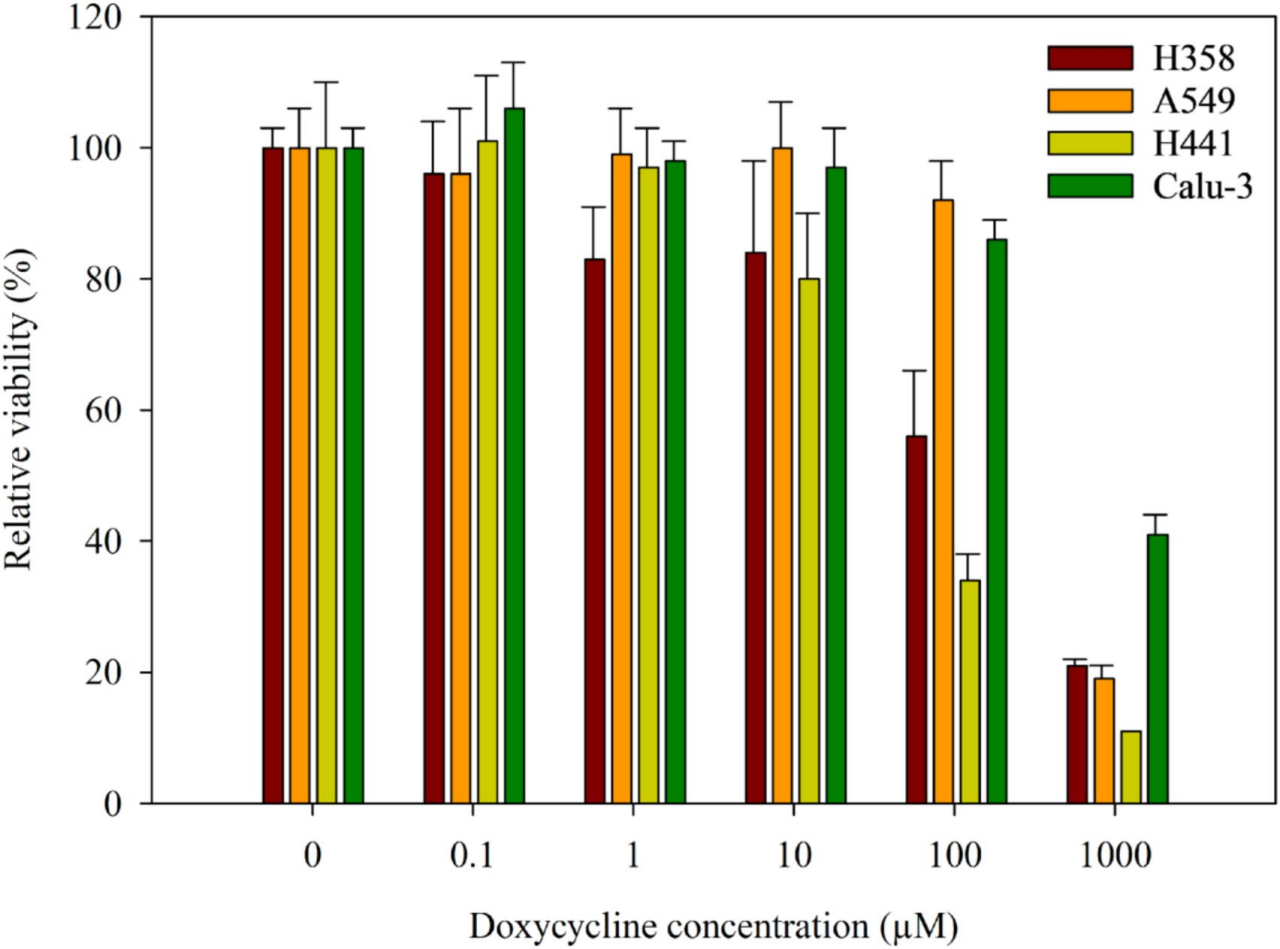
*In vitro* cytotoxicity response of raw Doxycycline on H358 cells, A549 cells, H441cells, and Calu-3 cells after 72 hours of exposure to different drug concentrations (n = 6, Mean ± SD).

As Figure 7 shows, at a drug dosage of 1,000 µM, there was a statistically significant decrease in cell viability (< 50% for all cell lines). In H441 cells, a 100 µM drug concentration reduced cell viability by <50%. Although 10 µM showed a statistically significant reduction in cell viability for H358 and H441 cells, viability was still >80%. Therefore, 10 µM was determined to be a safe concentration of the raw drug for the four cell lines.

## Discussion

While Doxycycline was spray dried previously, to our knowledge this study is the first time Doxycycline has been spraying dried with no excipients^31^For instance, in 2008, Gueders et al. successfully demonstrated that Doxycycline delivered as aerosol decreased airway inflammation caused by allergens through modulating cytokine production and MMP activities in mice^18^. While other studies used different carriers, such as carboxymethyl cellulose, sodium alginate, and leucine, to spray dry Doxycycline Hyclate. Our study offers several advantages such as cost-effectiveness, simplified formulation, and reproducibility^32^. SEM micrographs of raw drug and SD formulation showed the impact of the spray drying process on particle size and shape. Raw Doxycycline showed large needle-shaped particles unsuitable for inhalation and lung deposition. Moreover, particle size and shape were different between the three formulations. The SD powder particles at 100% and 50% PR appeared spherical with a crumpled surface and some degree of agglomeration, suggesting rapid particle drying. However, particles at 10% PR surface lacked the surface dimples seen in SD powders produced at the high and medium spray drying feed pump rates. All SD powders exhibited discrete particles. Raw Doxycycline crystalline powder had a low residual water content which is characteristic of crystals, as they exist in a lower energy state (i.e. low ΔG) with low molecular mobility in contrast to non-crystalline amorphous powders. The residual water content was higher than expected for dry powders intended for inhalation. Overall, the morphology, particle size, and water content were in the accepted range for inhalation. The XRPD patterns of raw Doxycycline showed intense and sharp peaks indicative of long-range molecular order suggesting a crystalline material. After spray drying, the SD powder’s diffraction pattern lacked any peaks suggesting an amorphous material. The crystallinity of the raw drug was further confirmed by DSC. A single main endothermic peak at 170 °C molecular order-to-disorder phase transition followed by an exothermic molecular disorder-to-order phase transition peak at 210°C followed by another molecular order-to- exothermic molecular disorder-to-order phase transition peak at 210°C and then another molecular order-to- disorder phase transition (i.e. solid-to-liquid melting) at 280°C. The detection of T_g_and ΔC_p_, which are second-order kinetic phase transition parameters characteristic of the amorphous solid-state, in all spray dried powders confirmed the creation of the amorphous powders following spray drying under these spray drying conditions. At temperatures below T_g_such as room and biological temperatures, the SD powders existed as amorphous glass. At temperatures above T_g_, the SD powders were in the amorphous rubbery phase. The presence of moisture in amorphous material significantly reduces glass transition temperature through plasticization, in which water molecules disrupt the intermolecular forces holding the powder together^18^. Our study shows that SD with higher water content exhibits lower T_g_^33^. HSM, ATR-FTIR and Raman spectroscopy were conducted to add complementary and confirmatory information to DSC and XRPD. In HSM, birefringence (i.e., a distinct characteristic of crystallinity) was visible in Doxycycline powder while the three SD powders lacked birefringence which confirms non-crystallinity. This aligns with the DSC and XRPD data demonstrating that the raw drug powder was in the crystalline solid-state, while the SD powders were shown to be in non-crystalline indicating that the spray drying process resulted in the loss of crystallinity. ATR-FTIR and Confocal Raman complement DSC and XRPD by providing information on intermolecular changes associated with different PR. In our study ATR-FTIR spectra showed the absence of peaks between 1400-1700 cm ^-1^. The broad peak at 3300 cm^-1^ was still present in the powders produced at 100% and 10% spray drying feed pump rates. However, the broad peak at 3200 cm^-1^ wasn’t present in the powder produced at the 50% spray drying feed pump rate indicating a feed pump rate effect on the molecular interactions in the resulting SD powders. The molecular interactions changed in the SD powders due to the spray drying process when compared to raw Doxycycline powder, as demonstrated by both ATR-FTIR and Raman spectroscopy molecular fingerprinting. Confocal Raman microscopy chemical imaging confirmed the composition homogeneity for each of the SD powders, as would be expected for a one-component powder system.

Cell viability confirmed safety across all the pulmonary cell types studied for most of the doses given. There was a difference in % cell viability among the cell types at the high 1,000 µM and 100 µM drug concentrations. As expected, the aerosol dispersion properties were influenced by the DPI device used which reflects the device-formulation interactions. NeoHaler gave good aerosol dispersion of the SD powders under the conditions studied, as seen in the aerodynamic parameters and NGI stage deposition patterns. Compared to other studies that spray dried Doxycycline Hyclate with excipients, our formulations had an aerodynamic diameter ranging from 3.07-6.71 μm compared to 3.74–6.54 μm and 3.11 μm. FPF in our formulation ranged from 19.6-36.72%. compared to 24.7-59.8, and 49.3%. However, as mentioned earlier, the water content in our formulation was inferior 6.4-6.69% vs .386-4.68% and 3.86% in the mentioned studies. It is important to mention that not only does the type of DPI contribute to the drug release from the device and lung deposition and the overall efficiency of delivery of the drug but also patient induced inspiratory effort^32^. A study conducted by Alman et al comparing Breezhaler^®^, Ellipta^®^ and HandiHaler^®^ showed that a patient with COPD needed less effort and higher peak inspiratory flow with Breezhaler^®^ compared to Ellipta® and HandiHaler. On the other hand, Handihaler didn’t show promising results. All SD powders displayed measurable powder deposition on the NGI stages with nanometer aerodynamic sizes, efficient emission out of both DPI devices giving high ED values, and high FPF values. The NeoHaler DPI device exhibited superior aerosolization properties of MMAD, FPF, and RF. Additionally, particles spray-dried at a high spray drying pump rate showed slightly improved performance compared to those powders produced at medium and low spray drying pump rates. While our formulation demonstrated good aerosol dispersion and lung deposition, future research should explore doxycycline formulations that include excipients like phospholipids and polymers. Further optimization of these formulations aims to enhance lung deposition and achieve ideal physicochemical properties for lung delivery.

## Conclusions

Excipient-free DPIs of Doxycycline were successfully developed using advanced spray drying in closed-mode conditions and reported for the first time. The SD particles produced had particle properties that are essential for pulmonary delivery as inhalation aerosols. All SD one-component drug powders were rendered amorphous as confirmed by the absence of long-range molecular order and detection of a measurable Tg and ΔCp. Molecular fingerprinting spectroscopy showed that the molecular interactions changed following spray drying. In addition, the molecular fingerprint spectra of the SD amorphous glass powders were different for each feed pump rate indicating differences in the molecular interactions occurring in the amorphous state for each type of SD powder. All SD powders readily aerosolized using two different FDA-approved human DPI devices. The formulation-device interactions were demonstrated to influence aerosol dispersion performance.

## Data Availability

The datasets used and/or analysed during the current study available from the corresponding author on reasonable request.

## Abbreviations

ATR-FTIR: Attenuated total reflectance-fourier-transform infrared spectroscopy
CRP: C-reactive protein
DPI: Dry powder inhaler
DSC: Differential scanning calorimetry
ED: Emitted dose
FDA: Food & drug administration
GSD: Geometric standard deviation
HSM: Hot stage microscopy
IL-6: Interleukin-6
KFT: Karl fischer titration
MMAD: Mass median aerodynamic diameter
MMP: Matrix metalloproteinase
OSA: Obstructive sleep apnea
RF: Respirable fraction
SD: Spray dried
SEM: Scanning electron microscopy
TNF-α: Tumor necrosis alpha
XRPD: X- Ray powder diffraction
%FPF: Fine particles emitted
